# A Systematic Review of Factors Affecting Mental Health and Well-Being of Asylum Seekers and Refugees in Germany

**DOI:** 10.3389/fpsyt.2021.643704

**Published:** 2021-03-18

**Authors:** Vivien L. Hajak, Srishti Sardana, Helen Verdeli, Simone Grimm

**Affiliations:** ^1^Department of Psychology, Medical School Berlin, Berlin, Germany; ^2^Global Mental Health Laboratory, Teachers College, Columbia University, New York, NY, United States; ^3^Department of Psychiatry, Charité, Campus Benjamin Franklin, Berlin, Germany

**Keywords:** asylum seekers, refugees, mental health, well-being, post-migration, Germany, contextual factors

## Abstract

**Background:** Since the onset of the 2015 European refugee crisis, ~4. 46 million people have sought asylum in the European Union, with Germany logging the largest share of all asylum applications. In addition to the severe adversities before and during flight, the process of settling into a new environment involves stressors that affect psychological well-being and mental health. The aim of this systematic review was to examine contextual factors during post-migration that influence the mental health and well-being of asylum seekers and refugees (ASRs) in Germany.

**Methods:** Following PRISMA guidelines, a systematic review was conducted across multiple databases for English and German studies published between 2015 and 2020 with index keywords.

**Results:** From a total of 303 articles, 156 duplicates were removed and, after title review, another 87 were excluded for not meeting the inclusion criteria. After assessing the abstracts of the remaining 60 articles, 39 were excluded. Full texts of 21 articles were assessed for eligibility and after excluding 8 articles, 13 articles were included in the review. The results demonstrate high rates of psychological distress among ASRs in Germany and the significant influence of contextual factors on their mental health and psychological well-being. The risk factors for poor mental health include an uncertain asylum status, living in shared asylum accommodations, separation from the nuclear family, lack of German language skills, integration issues and discrimination, while employment is a protective factor.

**Conclusion:** Asylum seekers and refugees have high prevalence rates of psychological distress directly influenced by contextual factors in Germany. Based on these findings, policy makers are strongly recommended to apply preventive strategies to reduce mental health problems of ASRs in Germany.

## Introduction

In recent years, Europe has been challenged by the largest migration wave since the end of the Second World War (1). The dramatic increase in the number of people seeking asylum in the European Union (EU) reached its historical climax with the European refugee crisis in 2015 with ~1.3 million applications, about twice as many as in 2014 (2–4). Between 2015 and 2019 ~4.46 million people have sought asylum in the EU (5).

According to the United Nations High Commissioner for Refugees (UNHCR), asylum seekers are individuals who are seeking international protection, but whose application for refugee status has not yet been determined (6). Once the asylum process has been completed, an asylum seeker who has received refugee protection is referred to as a refugee recognized under the Geneva Convention (7, 8). A refugee, according to Article 1 of the Geneva Convention, is a person who has a “well-founded fear of being persecuted for reasons of race, religion, nationality, membership of a particular social group or political opinion, is outside of the country of his nationality and is unable or, owing to such fear, is unwilling to avail himself of the protection of that country” [1951 Refugee Convention in (9)].

Compared to other European countries, in 2015 Germany hosted the majority of individuals who sought protection through asylum [around 476,510 asylum-applications; (2)]. The requests reached their peak in 2016 at 745,545, but steadily declined from 222,683 in 2017, to 185,853 in 2018 and to 165,938 in 2019 (10). Despite the decline in total asylum applications, Germany still logs the largest share of all EU asylum claims and continues to be the third largest recipient of new asylum claims worldwide (4, 11). At the same time, Germany takes in the highest number of refugees compared to its European neighbors (12). Based on data from the Central Register for Foreigners (Ausländerzentralregister, AZR), at the end of 2019, around 1.8 million refugees were living in Germany, 1.4 million of whom held a protection status (13). The overall protection rate for refugees (the rate of approved asylum applications) was 35% in 2018 and 38.2% in 2019 (10). According to German asylum law, a residence permit which allows a stay in the country is granted by recognition of refugee status, subsidiary protection or a ban on deportation (10, 14).

Traumatic events (TEs) experienced by asylum seekers prior to and during their flight involve unmet basic needs for survival, such as regular access to water and food, shelter and medicine; fearing for one's life, the death of a loved one, and forced separation from family; witnessing acts of violence, bombing and shooting, living in a war zone; imprisonment, and living in a refugee camp (15–17). Between 50 and 85% of asylum seekers and refugees (summarized abbreviation in this article as ASRs) report at least one TE (17, 18). Exposure to TEs is a major risk factor for the development of mental disorders such as post-traumatic stress disorder (PTSD), depression and anxiety disorders among others (19–24). Greater exposure to TEs leads to more pronounced symptoms of mental disorders, especially depression and anxiety disorders (21). ASRs are therefore classified as a vulnerable population at high risk for mental stress and mental disorders (18, 25, 26).

Given the number of ASRs living in Germany and the country's high level of resources for addressing mental health needs, it is crucial for policy makers and ASR advocates to have a clear understanding of ASRs' mental health needs and priorities. However, prevalence rates of mental disorders among ASRs in Germany are inconsistent (27). Studies report prevalence rates ranging from 21.7 to 57.1% for depression, 35 to 53.3% for anxiety disorders and 13 to 34.9% for PTSD (17, 28–32). A recent study showed that about half of asylum seekers already have at least one mental disorder upon arrival in Germany (17). In comparison with the prevalence rates of the German population (33), empirical evidence indicates significantly higher rates of mental disorders among ASRs (20, 29, 31, 34). The situation of ASRs has therefore been called a mental health crisis (35).

In addition to TEs before and during migration, adaptation to a new environment (post-migration) also includes potential socioeconomic, social and interpersonal stressors, as well as migration-related barriers to legal residence in the resettlement country, that have been associated with impaired psychological functioning and poorer mental health (22, 25, 36–38). In a study by Bogic et al. (39), post-migration stressors (PMSs) were directly related to mental disorders in long-settled war refugees. More migration-related stress as well as having only a temporary residence status were predictors for higher rates of mood and anxiety disorders and PTSD. Unemployment was associated with mood disorders, and not feeling accepted by the host population was associated with higher rates of both mood and anxiety disorders. Compared with Italy and the United Kingdom (UK), refugees in Germany not only had the highest prevalence rates of mental disorders, but also reported the highest number of PMSs. This needs further examination given the studies indicating that the influence of PMSs on psychopathology might be greater than that of pre-migration experiences (40, 41).

A substantial number of studies have addressed the effect of contextual factors on the mental health and well-being of ASRs in Germany during post-migration. The heterogeneity of variables examined makes it almost impossible to not only gain an overview but also identify significant associations between these factors. Furthermore, there has been no systematic review linking the different variables investigated in these studies. This paper aims to provide the first systematic review that identifies, synthesizes and evaluates the evidence of associations between post-migration contextual factors and mental health and well-being of ASRs in Germany.

## Methods

A systematic search of empirical original studies reporting on factors affecting the mental health and well-being of ASRs in Germany was performed for this review. Throughout this process, the PRISMA (Preferred Reporting Items for Systematic Reviews and Meta-Analyses) statement for conducting and reporting systematic reviews (42) was followed except pre-registration (Supplementary Table A1).

### Search Strategy

In March and October 2020, a systematic article search was carried out in the following medical and psychological electronic databases: APA PsycArticles (*via* Ebsco), APA PsycINFO (*via* Ebsco), CHINAL (*via* Ebsco), MEDLINE (*via* Ebsco), PubMed and PubPsych. All databases were searched using the following combination of keywords: (“factors^*^” AND/OR “stressors^*^” AND/OR “mental health^*^” AND/OR “well-being^*^”) AND (“refugees^*^” AND/OR “asylum seekers^*^”) AND (“Germany^*^”). In order to focus the search on studies reporting on mental health and well-being of ASRs after their arrival in Germany, the term “post-migration” was partly added to the search terms. The search strategies were slightly modified for each database. All searches were limited to German and English language articles published between 2015 and 2020. Additionally, the reference lists of articles were parsed to identify further potentially relevant articles.

### Inclusion and Exclusion Criteria

Studies were included in the review if they: (i) investigated an adult sample of asylum seekers and/or refugees; (ii) reported on factors or quantitative estimates of mental health (e.g., depression, anxiety, PTSD) and/or reported on associative factors of well-being; (iii) were conducted in Germany, and (iv) were empirical original studies. Since international law makes a clear distinction between refugees who are forced to flee their country and migrants who voluntarily leave their country in search of better life prospects (43), only studies that examined asylum seekers and refugees were included. In accordance with the aim of this review, only articles reporting on contextual factors associated with mental health and well-being of ASRs in Germany were included. Contrary to the JBI (Joanna Briggs Institute) recommendation of limiting the search to literature published within the past 10 years (44), the detailed search strategy was instead restricted to literature which had been published within the past 5 years (since January 2015). This was done to better reflect the situation for ASRs in Germany since the beginning of the European refugee crisis. However, specific focus on a nationality was not an inclusion criterion. To obtain a comprehensive representation of current empirical knowledge and to increase the likelihood of including all essential information, there was no specification of the study design (e.g., cross-sectional, cohort) or on the study type. Thus, both quantitative and qualitative studies were included, given that the inclusion of qualitative studies in systematic reviews has become increasingly common to facilitate insight into current research (45).

Studies that provided data focusing exclusively on ASR children, adolescents or pregnant women were excluded. Due to their specific characteristics these most vulnerable subgroups within the ASR population would need to be examined separately. However, if a study examined adult ASRs together with these subgroups it was included in the review.

### Study Selection

The selection of studies was carried out in a three-step procedure: (1) after eliminating duplicates, titles were screened and non-applicable articles were excluded, (2) study abstracts were screened for inclusion and exclusion criteria, and (3) remaining studies were retrieved and reviewed as full text articles for eligibility in the review.

### Data Collection Process

A data key extraction sheet was developed including: (i) first author and publication year; (ii) study design; (iii) sample size; (iv) nationality; (v) gender; (vi) age; (vii) duration of stay in Germany; (viii) assessment instruments; (ix) mental health prevalence; (x) significant factors. The first author extracted the data from included studies. Disagreements were resolved by discussion between the first and the last review authors; if no agreement could be reached, it was envisaged that a third author would decide.

### Quality Assessment

A critical appraisal of the risk of bias in individual studies was used to evaluate the methodological quality of included studies in terms of design, conduct, and analysis. The methodological quality of included studies was assessed using the Joanna Briggs Institute (JBI) critical appraisal checklist for analytical cross-sectional studies, combined with a self-created question generated from the combination of three questions (items 8–10) of the JBI critical appraisal checklist for cohort studies to include assessment of follow-up procedures (46–48). The presence of each criterion was rated as “yes,” “no,” “unclear” or “not applicable” (46, 48, 49). The appraisal process was conducted by the first author, and, if any uncertainties were present, the articles were discussed with the last author until a consensus was reached. Articles were included in the review if they met the minimum quality of five criteria. The results of the quality assessment were used to describe the overall quality of the included studies and to score the quality of each individual study.

## Results

### Results of Search Strategy

**Figure 1:**
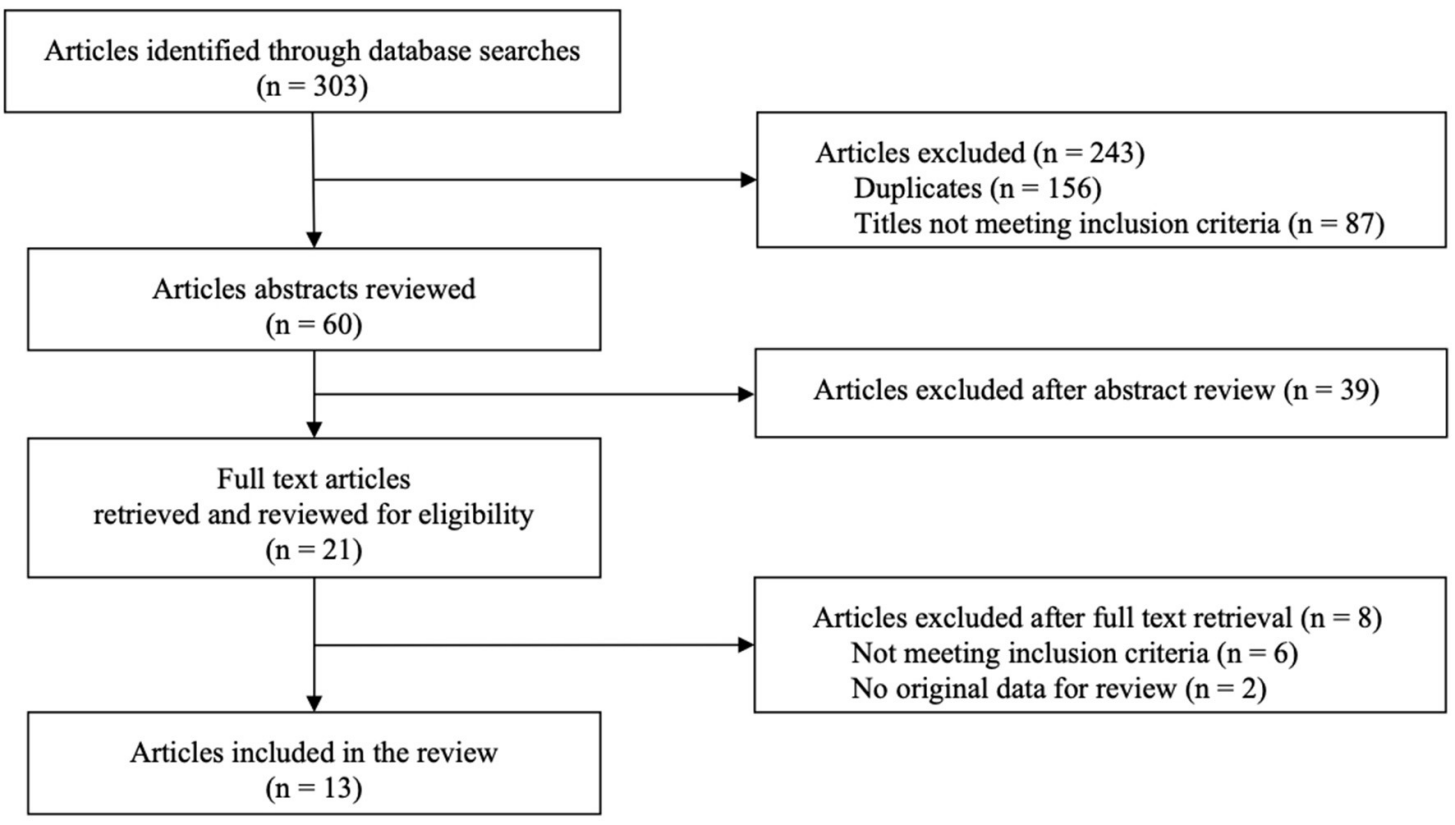
PRISMA flow chart of study selection process.

A total of 303 articles were identified through the systematic database searches. After removing duplicates (*n* = 156) and excluding articles whose titles did not meet the inclusion criteria (*n* = 87), the abstracts of 60 remaining articles were reviewed. The abstracts of 39 articles did not meet the inclusion criteria and thus were excluded. Full texts of the remaining 21 articles were retrieved and reviewed and another 8 articles were excluded: 6 for not meeting the inclusion criteria and 2 for not providing original data. The search thereby resulted in a total of 13 articles that met the inclusion criteria and thus were included in the systematic analysis. All included studies were published in English except for two which were published in German (50, 51). All relevant information has been translated into English. The summary of the search strategy is illustrated as a flow chart based on the PRISMA recommendation in Figure 1 (42).

### Descriptive Data of Included Studies

The studies were published between 2018 and 2020. The most commonly used study design was cross-sectional (50–59); two studies were longitudinal (60, 61) and one study used a mixed-methods design (62). All studies reported on ASRs, with the term “asylum seekers” or “refugees” being used as a general term for both groups, regardless of their specific legal status. Sample sizes ranged from 57 participants to 6,821 participants. Most studies included different ethnic groups such as Syrian, Afghan, Iraqi and others, and ethnic groups varied among studies. Four studies focused on Syrian refugees (52, 53, 61, 62), however, in all other studies the majority of ASRs were also Syrian. In all studies reporting gender distribution, the proportion of men was higher (60.3 to 85.1%), with the exception of one study with 60.0% female participants (60). The mean age of participants ranged from 28.46 years to 36.92 years (range 12–76 years). Three studies included children, one from the age of 12 (60) and two from the age of 14 (52, 55). The duration of stay in Germany varied between 1 day to nearly 6 years. A summary of the main characteristics of included studies is provided in Table 1.

**Table 1:** Characteristics of included studies.

**References**	**Study design**	**Sample size, *n***	**Nationality in %**	**Gender in %**	**Age in years, *M* (SD; range)**	**Duration of stay in Germany in months, *M* (SD; range)**	**Assessment instruments (mental health)**	**Mental health prevalence in %**	**Significant factors**
Borho et al. (61)	Longitudinal	108	Syrian	♀ 31.5♂ 68.5	T1: 35.67 (10.93; 19–63) T2: 36.92 (10.81; 20–64)	T1: 22.81 (6.90; 2–50)T2: 42.02 (6.35; 18–67)	PHQ-9 GAD-7 ETI	• DEP: 26.9 (T1), 30.6 (T2) • GAD: 16.7 (T1), 15.7 (T2) • PTSD: 13.9 (T1), 13.0 (T2) • ≥1 diagnosis: 31.5 (T1), 37.0 (T2)	• Asylum status • Discrimination
Comtesse and Rosner (54)	Cross-sectional	99	Arabic: 45.4 Kurdish: 32.3Afghan: 15.2Other	♀ 32.3♂ 67.8	30.12 (9.43; 19–74)	16.56 (12.92)	PHQ-9 PCL-5 TGI-SR	• DEP: 42.4 • PTSD: 45.4 • PGD: 20.2 • PCBD: 16.1	• Asylum status
El Khoury (52)	Cross-sectional	214	Syrian(Arabic: 86.4 Kurdish: 13.6)	♀ 39.7 ♂ 60.3	≥ 14	<10: 7.0%11–20: 9.3%21–30: 64.0%31–40: 14.5%> 41: 5.0%	MHI-18	• DEP: ♀ *M* = 14.47 (SD = 4.84) • ♂ *M* = 14.78 (SD = 4.66)	• Accommodation
Georgiadou et al. (53)	Cross-sectional	200	Syrian	♀ 30.5♂ 69.5	33.3 (10.6; 18–63)	23.3 (6.5; 2–52)	PHQ-9 GAD-7 ETI	• DEP: 14.5 • GAD: 13.5 • PTSD: 11.4 • ≥1 diagnosis: 30.5	• Asylum status
Grochtdreis et al. (56)	Cross-sectional	6,821	Syrian: 50.0Iraqi: 13.5Afghan: 13.4Other	♀ 38.9♂ 61.1	32.9 (10.78)	(12–48)	MCS	• *M* = 47.9	• Asylum status • Occupation
Haase et al. (55)	Cross-sectional	94	16 different(Syrian: 30.9)	♀ 14.9 ♂ 85.1	28.46 (9.58; 14–56)	11.78 (12.75; 1–50)	MIRIPS	• *M* = 2.35 (SD = 0.78)	• Accommodation • Family • Discrimination
Kaltenbach et al. (60)	Longitudinal	57	Syrian: 39.0Afghan: 26.0Iraqi: 7.0Other	♀ 60.0♂ 40.0	30.3 (11.5; 12–56)	9.3 (6.6; 2–36)	PHQ-9 PCL-5 PSS-I	• DEP: 16.0 (t0), 27.0 (t6), 16.0 (t12) • PTSD: 32.0 (t0), 27.0 (t6), 24.0 (t12)	• Occupation • PMSs
Löbel (57)	Cross-sectional	3,400	SyrianAfghanIraqiOther	Na	≥ 18	Na	MCS	• *M* = 49.17 (SD = 10.4; range 11.04–74.34)	• Asylum status • Accommodation • Family
Nutsch and Bozorgmehr (51)	Cross-sectional	4,465	Syrian: 49.0Iraqi: 12.9Afghan: 12.8Other	♀ 37.9♂ 62.1	33.6 (10.4)	Na	PHQ-2 GAD-2	• MD: 19.4 • GAD: 21.5	• Asylum status • Accommodation • Occupation
von Haumeder et al. (62)	Mixed-methods	127	Syrian	♀ 33.9♂ 66.1	31.9 (10.68; 18–67)	23.7 (10.39; 0.5–48)	PCL-5	• PTSD: 46.5	• Accommodation • Occupation • Discrimination
Walther et al. (58)	Cross-sectional	4,325	Syrian: 41.2Afghan: 13.9Iraqi: 8.8Other	♀ 26.5♂ 73.5	30.94 (10.47; 18–76)	1.35 years (0.71 years; 0–3.92 years)	PHQ-4	• Psych. distress: *M* = 3.14 (range 3.05–3.22)	• Asylum status • Accommodation • Occupation • Family • Language • Integration
Walther et al. (59)	Cross-sectional	2,569	Syrian: 44.2Afghan: 13.6Iraqi: 8.5Other	♀ 25.4♂ 74.6	≥ 18	Na	RHS-13	• Psych. distress: 41.2Mild: 19.7Moderate: 10.6Severe: 10.9	• Asylum status • Accommodation • Occupation • Family • Integration
Winkler et al. (50)	Cross-sectional	650	Syrian: 36.9 Afghan: 14.9Iraqi: 8.5Other	♀ 25.2♂ 74.8	30.6 (10.0)	128.3 days (177.3 days; 1–2,159 days)	HSCL-25 PDS HTQ SOMA-Scale	• DEP: 61.3 • GAD: 52.3 • PTSD: 41.7 • Somatic symptoms: 47.8 • Suicidal thoughts: 17.5 • ≥1 diagnosis: 74.6	• Asylum status • Accommodation • Integration

*Na, Not applicable; n, total sample size; M, mean; SD, standard deviation; DEP, Symptoms of depression; GAD, Symptoms of generalized anxiety disorder; MD, Symptoms of major depressive disorder; PCBD, Symptoms of persistent complex bereavement disorder; PDG, Symptoms of prolonged grief disorder; PTSD, Symptoms of post-traumatic stress disorder; PMS, Post-migration stressor; T1, Initial measurement; T2, Measurement after one and a half years; t0, Baseline measurement; t6, Measurement after 6 months; t12, Measurement after 12 months; ETI, Essen Trauma Inventory; GAD-2, Generalized Anxiety Disorder-2; GAD-7, Generalized Anxiety Disorder-7; HSCL-25, Hopkins-Symptom-Checklist 25; HTQ, Harvard Trauma Questionnaire; MHI-18, Mental Health Inventory; MIRIPS, Mutual Intercultural Relations in Plural Societies; MCS, Mental Health Component Summary Scale; PCL-5, Posttraumatic Stress Disorder Checklist-5; PHQ-2, Patient Health Questionnaire-2; PHQ-4, Patient Health Questionnaire for Depression and Anxiety; PHQ-9, Patient Health Questionnaire-9; PDS, Posttraumatic Diagnostic Scale; PSS-I, PTSD Symptom Scale—Interview Version; RHS-13, Refugee Health Screener–13; SOMA-Scale, SOMA-Scale of the Symtom-Checklist-90; TGI-SR, Traumatic Grief Inventory Self-Report Version*.

### Prevalence of Psychological Distress

All studies used diagnostic instruments to assess mental health symptoms. Seventeen different instruments were used in these studies. In six studies, questionnaires were completed by participants (self-report) (50, 52, 53, 55, 61, 62), while the studies by Comtesse and Rosner (54) and Kaltenbach et al. (60) used semi-structured clinical interviews, and five studies conducted computer-assisted face-to-face interviews (51, 56–59). All instruments used in the studies and their validity are presented in Supplementary Table A3.

#### Depressive Symptoms

Depressive symptoms were most frequently assessed with the depression module of the Patient Health Questionnaire [PHQ-9; (53, 54, 60, 61)]. The nine-item PHQ-9 measures the severity of depressive symptoms according to the Diagnostic and Statistical Manual of Mental Disorders (DSM-5) during the last past weeks on a 4-point frequency scale (0 = not at all, 3 = almost daily). The sum score ranges from 0 to 27, with a cut-off score above 10 determining clinically relevant depressive symptoms (63). Using the PHQ-9, depressive symptoms were found in 14.5 and 42.4% of the participants with females having more severe symptoms (53, 54). Kaltenbach et al. (60) utilized the PHQ-9 to assess depressive symptoms during the course of 1 year and found rates of 16.0% at first measurement, which increased to 27.0% after 6 months, but returned to the baseline rate of 16.0% after 1 year. In the study by Borho et al. (61) rates of depressive symptoms measured with PHQ-9, also showed no significant change over the course of one and a half years [26.9% (T1), 30.6% (T2)], however, females suffered significantly from more severe symptoms at the second measurement. Winkler et al. (50) found depressive symptoms in 61.3% of participants using the Hopkins Symptom Checklist 25 (HSCL-25), which is a clinical symptom checklist with 25 items used to measure the differentiated assessment of anxiety and depression as well as the resulting global mental stress (64, 65). The 2-item Patient Health Questionnaire [PHQ-2; (66)] was used in the study by Nutsch and Bozorgmehr (51), in which 19.4% of the participants showed symptoms of portable major depression (MD) during the last two weeks. El Khoury (52) used the 18-item version of the Mental Health Inventory [MHI; (67)] to evaluate the psychological experiences of participants within the past 4 weeks. On the depression subscale of the MHI-18, where higher scores from a total score of 0 to 100 indicate better mental health, females showed a depression mean score of 14.47 (SD = 4.84) and males that of 14.78 (SD = 4.66), indicating high levels of depressive symptoms.

#### Symptoms of Anxiety

Using the Generalized Anxiety Disorder Scale [GAD-7; (68)], clinically relevant symptoms of generalized anxiety disorder (GAD) were found in 13.5% of the participants, with females having a higher severity of symptoms (53). The GAD-7 is a seven-item questionnaire measuring the severity of symptoms of GAD during the past 2 weeks (68). Although Borho et al. (61) found that GAD symptom rates remained relatively stable over one and a half years [16.7% (T1), 15.7% (T2)] using the GAD-7, stronger GAD symptoms were significantly predicted by females gender at both measurements. Using the Generalized Anxiety Disorder-2 [GAD-2; (69)], portable GAD symptoms were found in 21.5% of the participants (51). In the study by Winkler et al. (50) 52.3% of the participants showed symptoms of GAD, which was assessed with the HSCL-25.

#### Symptoms of Post-traumatic Stress Disorder

The Post-traumatic Stress Disorder Checklist-5 [PCL-5; (70)], which assesses the 20 DSM-5 symptoms of PTSD was used in three studies (54, 60, 62). Prevalence rates of PTSD symptoms were 45.4 and 46.5% (54, 62). While Kaltenbach et al. (60), using the PCL-5, found a PTSD symptom rate of 32.0% at initial measurement, followed by 27.0% after 6 months and 24.0% after 1 year, the PTSD symptom rate in the study by Borho et al. (61), in which the Essen Trauma Inventory [ETI; (71)] was used, hardly changed from 13.9% at initial measurement to 13.0% after one and a half years, with women having more severe PTSD symptoms at the baseline measurement. The ETI consists of items assessing potentially traumatic events as well as PTSD symptoms according to the DSM-5 (71). Also with the ETI, 11.4% of participants screened positive for clinically relevant symptoms of PTSD (53). Winkler et al. (50) assessed symptoms of PTSD through combining the Post-traumatic Diagnostic Scale [PDS; (72)], which measures the 10 ICD-10 (International Statistical Classification of Diseases and Related Health Problems) criteria of PTSD, with the 25 items of the Harvard Trauma Questionnaire [HTQ; (73)] and found a rate of 41.7% among the participants.

#### Other Symptoms

Somatic symptoms were investigated in only one study with a prevalence rate of 47.8% using the SOMA-scale of the Symptom-Checklist-90 (50, 74). Using the 18-item Grief Inventory Self-Report version [TGI-SR; (75)], according to the criteria of Prigerson et al. (76) and the DSM-5, symptoms of prolonged grief disorder (PGD) were found in 20.2% of the participants and that of persistent complex bereavement disorder (PCBD) in 16.1% (54). Suicidal thoughts were found in 17.5% of the participants (50). Between 30.5 and 74.6% of the participants reported symptoms of at least one psychological diagnosis (50, 53, 61). After one and a half years, 37.0% of the participants were still screened positive for symptoms of at least one psychological diagnosis (61).

General psychological distress, including symptoms of depression, anxiety, and PTSD, was identified in 41.2% of participants using the 13-item Refugee Health Screener [RHS-13; (77, 78)], with 10.6% experiencing moderate psychological distress and 10.9% experiencing severe psychological distress (59). In the study by Walther et al. (58), participants reported a mean value of 3.14 on the Patient Health Questionnaire for Depression and Anxiety [PHQ-4; (79)], indicating mild psychological distress on average in the past 2 weeks which was higher than the mean score of 1.76 previously determined as the rate for the general German population (80). On the Mental Health Component Summary Scale (MCS), which indicates “a state of mental health well-being” with a total score from 0 to 100, with higher scores indicating better mental health, mean scores of 47.9 and 49.17 were recorded, indicating lower mental health on average, especially among females (M = 46.1), than in the host population (56, 57). Participants received a mean score of 2.35 (SD = 0.78) out of a total score between 1 and 5 on the Mutual Intercultural Relations in Plural Societies [MIRIPS; (81)] questionnaire, also indicating more mental health problems (55).

### Contextual Factors

Seven significant contextual factors related to mental health and well-being were extracted from the included articles: asylum status, accommodation, occupation, family, language, integration and discrimination.

#### Asylum Status

Nine studies reported that asylum status was a significant predictor of mental health or well-being of ASRs in Germany. In contrast, one study found no connection between depression or PTSD and asylum status (60).

Waiting for an asylum decision contributed significantly to the deterioration of mental health (57). Participants who were waiting for a decision on their initial asylum application or were in appeal against a rejected asylum application, had significantly higher levels of psychological distress, lower mental health-related quality of life (HrQoL) and lower life satisfaction than those who received a positive decision regarding their asylum application (56, 58). Simultaneously, waiting for an asylum decision was associated with higher symptom rates of PGD (20.7% first application, 37.5% in appeal), PCBD (20.7% first application, 25.0% in appeal) and PTSD (55.1% first application, 59.3% in appeal) compared to having already received a residence permit (54). Participants with a pending or rejected application had a 1.76 times (range 1.52–2.05) higher risk of showing depressive symptoms than those with a recognized asylum application (51). Thereby, the subjective feeling of having waited a long time for an asylum hearing, regardless of the actual length of stay in Germany, was significantly associated with depressive symptoms (50). The chances of depressive symptoms were lower when the official hearing had already taken place (51). There was also a significant connection between depressive, GAD and PTSD symptoms and the participants' impression that not all asylum-relevant details were being communicated at the hearing (50).

Compared to participants who were waiting for an asylum decision or who were in appeal against it, those with a temporary residence permit were significantly more likely to report depressive symptoms (57.8%) but also reported lower PGD (5.2%) or PTSD (26.3%) symptom levels (54). However, a shorter duration of the residence permit's validity associated with report of more severe PTSD symptoms (53, 61). Similarly, a temporary residence permit was associated with more impairments due to PTSD symptoms compared to a temporary suspension of deportation or a border crossing certificate, but with less somatic symptoms (50).

The uncertain legal status of a subsidiary protection or a temporary suspension from deportation, both residence permits for 1 year, was significantly associated with report of psychological distress compared to participants with a granted residence status, with males reporting greater psychological distress (58, 59). Compared to a subsidiary protection, a temporary suspension from deportation was also associated with lower mental HrQoL (56).

Participants who received a border crossing certificate and were therefore directly threatened by deportation, reported significantly higher mean values of depressive symptoms than those with a temporary suspension of deportation or any other status (50).

#### Accommodation

Eight studies established a significant association between mental health or well-being of ASRs and their accommodation, while two studies found no such association (53, 60).

The quality of residence contributed up to 20.0% toward mental health (52) and, at the same time, the probability of depressive symptoms decreased with increasing housing satisfaction (51).

In a study using mix-methods, participants described housing issues to be the most challenging. Of all the participants reporting PTSD symptoms, only 26.5% were satisfied with their current housing situation, compared to 73.5% of those without PTSD. Simultaneously, only 35.0% of participants reporting PTSD symptoms confirmed that they had “enough food to eat” (compared to 65.0% of participants without PTSD) and 30.2% confirmed that they had “enough money to function well on a daily basis” (compared to 69.8% without PTSD). Therefore, the participants' unmet need for housing, food and sufficient money for essential daily expenses were associated with endorsement of PTSD symptoms (62).

Compared to living in private accommodations, living in shared accommodations proved to be a significant PMS associated with deteriorating mental health (57). Living in shared asylum accommodations was associated with an increase in reported psychological distress (55, 59). Additionally, refugees living in asylum centers experienced more instances of discrimination than those living in independent apartments, which, in turn, also contributed significantly to psychological problems (55) (see section Discrimination).

Conversely, refugees living in private or independent accommodations had better mental health as indicated by higher mental health scores on the MHI-18 (52), significantly lower levels of psychological distress and higher levels of life satisfaction than those living in shared refugee housing facilities (58).

Winkler et al. (50) pursued a more differentiated approach to different types of accommodations. Participants living in emergency accommodations such as schools, large aircraft halls or gymnasiums reported significantly more symptoms of depression, anxiety and PTSD, while the subjective quality of life was perceived as better in established initial reception centers and shared accommodations with small rooms or flats, both of which had experienced social work staff.

#### Occupation

While two studies (52, 57) found no significant effect of occupation on the mental health and well-being of ASRs, six studies found a significant impact.

The failure to meet employment needs was associated with PTSD symptom report as only 29.5% of participants with PTSD confirmed that they had access to education, skills training or employment programs compared to 70.5% of participants without PTSD. In the qualitative results, participants reported that unemployment, or employment below their occupational level led to “lower self-esteem, frustration and despair” (62).

A regular occupation such as work, school or apprenticeship was associated with lower psychological distress (60). Employed participants had significantly higher MCS scores indicating better mental health than unemployed participants and vice versa (56). The risk of depression was also almost twice as high among unemployed participants compared to those in employment (51).

A gender-specific effect showed that employment was linked to a reduction of psychological distress only among employed men (58). At the same time, psychologically distressed men had a strongly reduced probability of being employed or participating in educational programs. Psychologically distressed females, in particular, were less likely to participate in educational programs (59).

#### Family

Four studies identified the significance of family relationships with mental health and well-being of ASRs in Germany. A study by Haase et al. (55) showed that living alone was associated with more psychological problems compared to living with a family. Similarly, the study by Löbel (57) showed deterioration of mental health if the nuclear family (a partner or at least one minor child) did not live in the same location in Germany. This effect was even more pronounced if all members of the nuclear family lived abroad. If an adult child lived somewhere in Germany, the parents' mental health was lightly better compared to the mental health of parents whose children did not reside in Germany (57). Men who lived in Germany without their nuclear family were ~1.34 times more likely to report psychological distress than those who had their complete nuclear family in Germany (59). When seeking family reunion, men also showed higher levels of psychological distress and lower life satisfaction (58).

#### Language

Although studies by El Khoury (52) and Nutsch and Bozorgmehr (51) found that a command of the German language had no significant impact on mental health, they mentioned that many participants felt that learning German was the greatest obstacle to adapting to life in Germany. The only quantitative results from Walther et al. (58) associated better German language skills with lower levels of psychological distress and increased life satisfaction, especially among men.

#### Integration

The occurrence of psychological symptoms correlated significantly with lower attendance of German language courses, lower participation in sports activities, worse orientation to the surroundings, as well as a more pronounced sense of lacking support and feeling like a stranger (50). Similarly, for men, participation in integration courses was associated with a lower degree of psychological distress, although this effect was small (59). In contrast, lower levels of psychological distress and higher levels of life satisfaction among females were associated with the length of time spent with German natives (58). Conversely, other studies did not confirm that the interaction of participants with their host society (55), the number of German friends (52) or the context of reception (perceived kindness, feeling welcome, opportunities within the host country) (55) were predictors of mental health. The study by Nutsch and Bozorgmehr (51) showed that with increased loneliness, the chance of depressive symptoms also increased overall.

#### Discrimination

A significant association between discrimination and PTSD was found by one study where only 29.5% of participants with PTSD felt like they were treated fairly in their new community, compared to 73.5% of participants without PTSD. Confrontations with stereotypes and discrimination were also mentioned as being both frustrating and an obstacle to positive psychological adaptation (62). The level of perceived discrimination was also associated with psychological distress, particularly depressive and GAD symptoms (55, 61). One study did not find a relationship between the perception of discrimination by the host society and the mental health of ASRs (52).

#### Cumulative Post-migration Stressors

Overall, participants who experienced more PMSs showed an increase in PTSD symptoms and vice versa (60).

#### Quality/Risk of Bias

The results of the assessment of methodological quality for each study are provided in Supplementary Table A2. Overall, all studies met the minimum methodological quality and were therefore included in the review. Three studies were assigned to be at risk of selection bias within the study sample due to differences between responders and non-responders. Responders were older (53, 56), although in one study this was only the case at the second measurement (61), more often had Syrian nationality (56). Non-responders, on the other hand, had higher levels of psychological distress, such as high severity of PTSD symptoms (53, 61). Similarly, prevalence rates of mental health symptoms might have been underestimated. The sampling procedure in the IAB-BAMF-SOEP survey, whose data was used in three studies, might have resulted in a high risk of selection bias in favor of participants with higher levels of mental health and well-being, as is generally expected in population-based surveys (57–59). In two studies, subjects with more severe mental health symptoms were even completely excluded from study participation (50, 60). The lack of availability of survey instruments in all required languages might also have led to selection bias, as subjects who did not understand any of these languages were excluded (50, 51, 55). In addition, the results might have been biased by an unequal distribution of a variable within the study sample, such as a larger number of study participants residing in refugee establishments (52). All studies identified confounding factors such as gender, age or country of origin, but only five stated strategies to deal with these (52, 57–59, 61) and thus confounding biases could affect the results of the remaining eight studies. In general, all studies used validated instruments to assess psychological symptoms and appropriate statistical methods to determine outcomes. However, the self-report measures used in six studies (50, 52, 53, 55, 61, 62) might have biased the reported prevalence rates of psychological symptoms through effects of social desirability as one example (61). Similarly, respondents' answering behavior in semi-structured clinical interviews (54, 60) and computer-assisted face-to-face interviews (51, 56–59) might have been biased by social desirability, an assumed connection to the asylum hearings or the interviewer or interpreter. The study samples of three studies (51, 54, 61) had good comparability of characteristic with the general refugee population in Germany and were therefore representative. In contrast, conditions in the study samples of three studies (52, 55, 62) were not fully representative, implying limited generalizability to the lager population of refugees. Nevertheless, the results provide valuable insight into the current situation of the refugee population in Germany. Overall, the quality of the studies is adequate, but the heterogeneity of outcome measures across studies might display a risk of bias. Furthermore, the keywords and search terms might not have identified all relevant available data on the topic, which could represent an evidence selection bias.

## Discussion

This is the first systematic review demonstrating the significant influence of contextual factors during post-migration on the mental health and well-being of ASRs in Germany since the beginning of the European refugee crisis. Seven key contextual factors that are significantly associated with mental health and well-being of ASRs were extracted from the 13 articles included: Asylum status, accommodation, occupation, family, language, integration and discrimination.

### Prevalence of Psychological Distress

As confirmed in several other countries (20, 22, 82, 83) and by a recent systematic review and meta-analysis (84), the prevalence rates of psychological distress, particularly depressive symptoms (14.5 to 61.3%), GAD symptoms (13.5 to 52.3%) and PTSD symptoms (11.4 to 46.5%) are heterogeneous among the ASRs in this review, but high and persistent over time compared to the German population (33). The discrepancies found in this review could be explained by the use of a variety of 17 assessment instruments or by the different living conditions of ASRs that emerge as risk or protective factors. The included study by Georgiadou et al. (53) found comparatively low prevalence rates of depressive (14.5%), GAD (13.5%) and PTSD symptoms (11.4%) which might be due to the fact that only moderate to severe levels of depressive and GAD symptoms were described. These low prevalence rates were also seen as confirming the protective influence of positive contextual factors as all refugees in this sample lived in good conditions.

The persistently high prevalence rates of psychological distress present among ASRs in Germany raise the pressure for early professional psychosocial support and specific intervention programs for mental health issues. Although Germany is internationally obligated to provide medical assistance to asylum seekers immediately upon arrival according to §4 Asylum Seekers Benefits Act (AsylbLG), asylum seekers have only a limited entitlement to “necessary” treatment for “acute illnesses and pain illnesses and pain conditions” during the first 18 months of their stay (85, 86). While psychiatric treatment is part of acute care, psychotherapy is generally not included in the scope of benefits under the Asylum Seekers Benefits Act (AsylbLG) and must therefore be approved by the social authority (87). In addition to these legal barriers, several other administrative, linguistic and cultural barriers [e.g., language difficulties, delayed or missing cost coverage for language mediation, bureaucratic hurdles, increased costs, limited care capacities, long waiting times, (asylum-related) interruption of diagnostic and therapeutic processes, discrimination] impede access to adequate psychotherapeutic care provided for asylum seekers in Germany (86, 88). Overall, asylum seekers report poorer health compared to data from the German population, but, at the same, time they report lower utilization of health care, which may bring the influence of contextual factors even more to the fore (89).

### Asylum Status

Asylum status proves to be one of the most pressing post-migration stressors for the mental health and well-being of ASRs in Germany, thereby confirming several previous studies (83, 90). The mean duration of asylum procedures of initial and follow-up applications in Germany is 8 months (91). During this time, asylum seekers have to cope with uncertainty about the outcome of intensive legal proceedings over which they have little to no control and which are, at the same time, essential for their integration and future life (92). This presents an additional burden on asylum seekers during the migration phase in all countries worldwide (25). The extended duration of asylum procedures has been shown to significantly deteriorate mental health and lower the quality of life of asylum seekers (19, 93–95), which is confirmed by the results of this review. Compared to holding a residence permit, waiting for an asylum decision is associated with higher levels of depressive, PTSD, PGD, and PCBD symptoms as well as lower mental HrQoL and lower life satisfaction. The study by Silove et al. (96) showed that symptoms of depression, PTSD, anxiety and mental health functioning improved in asylum seekers who had been granted a residence permit. While we show that a temporary residence permit is significantly associated with higher rates of depressive symptoms, it is, however, associated with less severe PTSD and PGD symptoms. A longer duration of the residence permit is associated with less severe PTSD symptoms. In contrast, compared to a temporary suspension of deportation or a border crossing certificate, a temporary residence permit is associated with more impairment due to PTSD symptoms. This is in line with the results of Steel et al. (97) who found that temporary protection, as well as its validity contribute to greater stress due to the fear of repatriation and the persistence of psychiatric disorders such as depression, PTSD and mental health-related disabilities in Arab-speaking refugees from Mandaean in Sydney. Bogic et al. (39) complement these findings by linking temporary residence status to higher rates of mood and anxiety disorders. When temporary residence status was changed to permanent residence status, the study by Nickerson et al. (98) showed an improvement in depression, PTSD symptoms and quality of life. In this current review, compared to a granted residence status, the uncertain subsidiary protection or temporary suspension from deportation is associated with psychological distress, with men being more distressed. A suspension from deportation also predicts a lower mental HrQoL. Refugees who received a border crossing certificate have significantly higher mean values of depressive symptoms. Along that line, Raghavan et al. (99) found that receiving asylum status, and therefore a secure legal status, had the greatest effect in reducing the severity of symptom among asylum seekers. All these findings contrast to the study by Kaltenbach et al. (60), included in this review, which found no significant evidence for an association between PTSD and depression with asylum status at the first measurement. However, because the majority of refugees in the study by Kaltenbach et al. (60) were still in the first stage of their asylum application, it is plausible that this status did not yet affect their mental health.

### Accommodation

Another major determinant of mental health and well-being of ARSs in Germany is accommodation, as highlighted by the included study by El Khoury (52), which estimated its contribution to mental health at up to 20.0%. ASRs living in emergency accommodations report significantly more frequent symptoms of depression, GAD and PTSD, while the subjective quality of life is perceived as better in established initial reception centers and shared accommodations. While living in a shared asylum accommodation proves to be a significant PMS deteriorating mental health, especially among men, and to be linked to more received discrimination, living in a private or independent accommodation is associated with better mental health and higher life satisfaction. Overall, the more satisfied ASRs are with their housing, the less likely they are to show symptoms of depression and PTSD. Although no conclusions can be drawn from the study by Richter et al. (100), it proved that the majority of refugees (63.6%) living in a Bavarian central refugee reception facility were suffering from one or more psychiatric disorders. International research largely confirms that living in shared institutions is connected to poor mental health amongst refugees (101). A systematic review and meta-analysis by Steel et al. (21), which included 40 countries, showed that refugees who were living in refugee camps had higher rates of depression than those resettled to high-income countries. Compared to Yugoslav refugees living in Italy and the UK, those living in Germany experienced the highest number of PMSs, including inadequate accommodation (39). The meta-analysis by Porter and Haslam (40) both confirms and expands these findings by concluding that refugees living in permanent private accommodation have significantly better mental health than those living in institutional and temporary accommodation. In two included studies, no correlation between mental health and living situation was found, which could be due to the fact that one had a comparatively small sample size (53) and the other sampled refugees who mostly lived in good conditions (60).

### Occupation

The current review offers indication that occupation is a protective factor for mental health and well-being in ASRs. Occupation such as work, school or apprenticeship is significantly associated with fewer depressive and PTSD symptoms and better overall mental HrQoL. International research confirms and complements these findings by linking unemployment with higher rates of mental disorders (19, 22, 37, 39, 102–105). This review also shows that employment status has a particular impact on the mental health of men, as those who suffer from psychological distress are less likely to be employed and, conversely, those who are employed have less psychological distress. Distressed men and women in particular are also less likely to participate in educational programs. Similarly, unemployment has been found to be a strong risk factor for the development of depression in males (102). A meta-analysis summarized that restricted work rights and employment prospects leading to restricted access to economic opportunities (106) deteriorate mental health in refugees world-wide (40). In contrast, a recently published study by Jannesari et al. (90) only found a weak positive association between unemployment and mental disorders.

### Family

Separation from the family is identified as a predictor of mental health deterioration among ASRs by the current review. Men who live in Germany without their nuclear family are 1.34 times more likely to experience psychological distress and lower life satisfaction if they are seeking family reunion. The negative effects of family separation on mental health have been demonstrated in a number of international studies where refugees isolated from their families living abroad were more likely to report psychiatric disorders such as depression and PTSD (97, 103, 104). Our findings are strengthened by the results of a study of Latin American and African refugees in Canada by Rousseau et al. (107), who found that seeking family reunification mitigates the link between past personal trauma exposures and psychological suffering. There are a number of hypotheses on what makes the separation from the family so critical. A study of Mandaean refugees from Iraq living in Sydney showed that not only the separation from the family, but also fearing for the safety of family members who were still living in their country of origin, was correlated with higher levels of depression, PTSD and increased mental health disability (108).

### Language

Integration into a host country also depends on social aspects that appear essential for adapting to life in Germany. Steel et al. (103) found poor host language proficiency to be one of the most significant risk factors for mental disorders. Similarly, this review indicates that better German language skills reduce the level of psychological distress and increase life satisfaction, especially among men. In a longitudinal study in Canada, the lack of English language skills had no influence on the mental health of Southeast Asian refugees at first, but it was a significant predictor for depression after a period of 10 years. Contrary to the German data in this review, this was particularly seen in women. This effect may be due to the fact that women were less likely to receive English language training and were therefore at higher risk of isolation (102). A systematic literature review associated poor post-migration economic factors, including a poor host language proficiency, with depression (22). Language difficulties also constituted a barrier to accessing mental health and psychosocial support (MHPSS) services (109).

### Integration

It is highly likely, but not proven, that proper language skills may influence the quality of contact between ARSs and the German host population. The current review shows that the deciding factor is not how many German friends ARSs have, but rather how much time they actually spend with the German host society. Spending more time with German natives is significantly associated with higher levels of life satisfaction and lower levels of psychological distress, particularly among women. Also, less participation in activities such as German language courses or sports is associated with more psychological symptoms. A study by Silove et al. (19) showed that social isolation, described as loneliness among refugees, is linked with depression, anxiety and PTSD. Even after more than 10 years of residence in Switzerland, psychological symptoms among refugees were associated with poor integration (110), which emphasizes the importance of this factor, especially with regard to long-term integration.

### Discrimination

At the same time, social integration depends on the acceptance of the ASRs' demands for equal rights. The acceptance of ASRs in Germany is markedly influenced by discrimination when one takes into account the society's irrational fears regarding people of different color and culture (111). The current review demonstrates that depressive, GAD and PTSD symptoms are associated with increased discrimination experiences by ASRs. This association was also shown by Kim (105), with refugees who experienced discrimination on a daily basis being more likely to suffer from depression. In the included study by El Khoury (52), only a small percentage of participants actually experienced discrimination, which may have resulted in no association found between discrimination and mental health.

### Limitations

Despite the significance of the data analyzed in this review, a number of limitations should be acknowledged when interpreting our findings. The review was not pre-registered. Furthermore, based on the search terms defined here, it cannot be excluded that all relevant studies were found, which, in turn, might have biased the review results. The included studies differed greatly in terms of sample size, nationalities of included participants, duration of stay in Germany and the instruments used. This heterogeneity, for specific subgroups in particular, made it difficult to put the studies into a valid and reliable context. Therefore, vulnerable subgroups of ASRs such as children, adolescents or pregnant women were excluded from the review. In a study conducted in Berlin, 7.0% of 164 refugee women surveyed were pregnant (112), but all researchers agreed that there is a major knowledge gap regarding research on this group (88). According to statistics from January to March 2020, minors accounted for about half of the asylum seekers in Germany (113). Consequently, the exclusion of these subgroups limits the generalizability of the published results to the entire ASRs population in Germany and requires particular attention in future research. Furthermore, the attempt to identify gender-specific differences could only be fulfilled to a limited extent due to unexamined interactions between PMSs and sex in the included studies. In addition, while women have been seen as more vulnerable in other studies on ASRs (97, 103, 104), the proportion of male participants was predominant in all included investigations, except for one. Even though this gender imbalance is representative for the German ASR population (10), it could influence the outcomes and cause distortions as gender differences in mental disorders have been identified *per-se* (114) and among ASRs in earlier literature (22, 115). Furthermore, we did not analyze the extent to which mental health outcomes can be associated with the nationality of ASRs, although different nationalities were examined as an independent variable in some studies. The focus on the Syrian refugee population in some studies could be explained by the fact that this group constitutes the largest one in Germany (113, 116). In order to successfully prevent the development or manifestation of psychological distress among ASRs in Germany, the development of culturally sensitive prevention measures could be crucial, and this aspect should therefore be given careful attention in future research. Another question that arises when interpreting the current results is what influence the duration of stay in Germany, which varied widely across the included studies, has on mental health. For example, Richter et al. (31) observed an increase in the prevalence of PTSD among asylum seekers studied in Bavaria between the initial measurement and the follow-up measurement 6 months later, with no evidence of additional traumatic events. Although the included study by Walther et al. (58) found that psychological distress decreased and life satisfaction increased with longer time stayed in Germany, this could not be confirmed by the majority of the included studies. The primary weakness of the included studies lies in the use of different instruments to assess mental health which, in particular, accounts for rather different prevalence rates of the identified mental disorders. Also, the method of data collection in the included data could make results questionable as they could be influenced by social desirability effects, language difficulties or different cultural understandings of terms describing psychological symptoms and therefore might lead to incorrect responses in the questionnaires and interviews. Moreover, due to their cross-sectional design, the majority of the study results do not allow for proof of causality and valid conclusions regarding the direct and causal relationship between post-migration factors and mental health of the surveyed population.

## Conclusion

This systematic review of the current literature demonstrates that ASRs in Germany are at high risk for mental health symptoms and lowered levels of well-being, both of which are associated with contextual factors. Post-migration stressors include uncertainties during the asylum process, living in shared asylum accommodations, separation from the family, poor German language skills and a lack of integration and discrimination which all contribute to the deterioration of mental health. In contrast, occupation is a protective factor. The findings thereby suggest the implementation of standardized and careful psychiatric screening of all representative groups of ASRs upon arrival in Germany and, as recommended by Richter et al. (31), repeated measurements after a certain period of time to better identify the validity and specificity of causal and coping factors. In line with the World Health Organizations demand (117), policy makers should use preventive strategies to improve mental health and well-being. These activities should consist of better structured asylum procedures, decentralized accommodation, improved access to the labor market, reunion of nuclear family members, offering more and individual language courses, improving contact with German nationals and anti-discrimination programs for ASRs as well as for the host population.

## Author Contributions

VH performed the systematic literature research, article selection, data extraction and created the first draft of the manuscript including the introduction, methods, results, and discussion. SG gave feedback and made essential corrections. HV and SS suggested crucial improvements. All of the authors contributed to the final manuscript and submission.

## Conflict of Interest

The authors declare that the research was conducted in the absence of any commercial or financial relationships that could be construed as a potential conflict of interest.
