# Supplementary material for: A Systematic Review of Factors Affecting Mental Health and Well-Being of Asylum Seekers and Refugees in Germany
Source: Front Psychiatry. 2021 Mar 18;12:643704. doi: 10.3389/fpsyt.2021.643704 (PMC8012840; doi:10.3389/fpsyt.2021.643704)
Supplement: Supplementary file 1 [file Data_Sheet_1.docx]

**Supplementary Table A1**. PRISMA checklist.

| **Section/topic** | **#** | **Checklist item** | **Reported on page #** |
| --- | --- | --- | --- |
| **TITLE** | | |  |
| Title | 1 | Identify the report as a systematic review, meta-analysis, or both. | 1 |
| **ABSTRACT** | | |  |
| Structured summary | 2 | Provide a structured summary including, as applicable: background; objectives; data sources; study eligibility criteria, participants, and interventions; study appraisal and synthesis methods; results; limitations; conclusions and implications of key findings; systematic review registration number. | 1 |
| **INTRODUCTION** | | |  |
| Rationale | 3 | Describe the rationale for the review in the context of what is already known. | 2-3 |
| Objectives | 4 | Provide an explicit statement of questions being addressed with reference to participants, interventions, comparisons, outcomes, and study design (PICOS). | 3 |
| **METHODS** | | |  |
| Protocol and registration | 5 | Indicate if a review protocol exists, if and where it can be accessed (e.g., Web address), and, if available, provide registration information including registration number. | N |
| Eligibility criteria | 6 | Specify study characteristics (e.g., PICOS, length of follow-up) and report characteristics (e.g., years considered, language, publication status) used as criteria for eligibility, giving rationale. | 3-4 |
| Information sources | 7 | Describe all information sources (e.g., databases with dates of coverage, contact with study authors to identify additional studies) in the search and date last searched. | 3 |
| Search | 8 | Present full electronic search strategy for at least one database, including any limits used, such that it could be repeated. | 3 |
| Study selection | 9 | State the process for selecting studies (i.e., screening, eligibility, included in systematic review, and, if applicable, included in the meta-analysis). | 4 |
| Data collection process | 10 | Describe method of data extraction from reports (e.g., piloted forms, independently, in duplicate) and any processes for obtaining and confirming data from investigators. | 4 |
| Data items | 11 | List and define all variables for which data were sought (e.g., PICOS, funding sources) and any assumptions and simplifications made. | 3 |
| Risk of bias in individual studies | 12 | Describe methods used for assessing risk of bias of individual studies (including specification of whether this was done at the study or outcome level), and how this information is to be used in any data synthesis. | 4 |
| Summary measures | 13 | State the principal summary measures (e.g., risk ratio, difference in means). | Na |
| Synthesis of results | 14 | Describe the methods of handling data and combining results of studies, if done, including measures of consistency (e.g., I^2^) for each meta-analysis. | Na |

| **Section/topic** | **#** | **Checklist item** | **Reported on page #** |
| --- | --- | --- | --- |
| Risk of bias across studies | 15 | Specify any assessment of risk of bias that may affect the cumulative evidence (e.g., publication bias, selective reporting within studies). | 4 |
| Additional analyses | 16 | Describe methods of additional analyses (e.g., sensitivity or subgroup analyses, meta-regression), if done, indicating which were pre-specified. | Na |
| **RESULTS** | | |  |
| Study selection | 17 | Give numbers of studies screened, assessed for eligibility, and included in the review, with reasons for exclusions at each stage, ideally with a flow diagram. | 5 |
| Study characteristics | 18 | For each study, present characteristics for which data were extracted (e.g., study size, PICOS, follow-up period) and provide the citations. | 5-10, Tab. 1 |
| Risk of bias within studies | 19 | Present data on risk of bias of each study and, if available, any outcome level assessment (see item 12). | 10-11 |
| Results of individual studies | 20 | For all outcomes considered (benefits or harms), present, for each study: (a) simple summary data for each intervention group (b) effect estimates and confidence intervals, ideally with a forest plot. | 5-10 |
| Synthesis of results | 21 | Present results of each meta-analysis done, including confidence intervals and measures of consistency. | Na |
| Risk of bias across studies | 22 | Present results of any assessment of risk of bias across studies (see Item 15). | 11 |
| Additional analysis | 23 | Give results of additional analyses, if done (e.g., sensitivity or subgroup analyses, meta-regression [see Item 16]). | Na |
| **DISCUSSION** | | |  |
| Summary of evidence | 24 | Summarize the main findings including the strength of evidence for each main outcome; consider their relevance to key groups (e.g., healthcare providers, users, and policy makers). | 11-14 |
| Limitations | 25 | Discuss limitations at study and outcome level (e.g., risk of bias), and at review-level (e.g., incomplete retrieval of identified research, reporting bias). | 14-15 |
| Conclusions | 26 | Provide a general interpretation of the results in the context of other evidence, and implications for future research. | 15 |
| **FUNDING** | | |  |
| Funding | 27 | Describe sources of funding for the systematic review and other support (e.g., supply of data); role of funders for the systematic review. | 18 |

*Note: N* = No; *Na* = Not applicable*; From: Moher D, Liberati A, Tetzlaff J, Altman DG, The PRISMA Group. Preferred Reporting Items for Systematic Reviews and Meta-Analyses: The PRISMA Statement. PLoS Medicine (2009). 6(7):e1000097. doi: 10.1371/journal.pmed.1000097*

**Supplementary Table A2**. Results of quality appraisal of included studies using the JBI checklist.

*Note: Y* = Yes, *N* = No, *U* = Unclear, *Na* = Not applicable

| **First Author (year)** | **1. Were the criteria for inclusion in the sample clearly defined?** | **2. Were the study subjects and the setting described in detail?** | **3. Was the exposure measured in a valid and reliable way?** | **4. Were objective, standard criteria used for measurement of the condition?** | **5. Were confounding factors identified?** | **6. Were strategies to deal with confounding factors stated?** | **7. Were the outcomes measured in a valid and reliable way?** | **8.**  **Was the follow up time reported, completed, and if not, were the reasons and strategies to incomplete follow up described and explored?** | **9. Was appropriate statistical analysis used?** | **Overall** |
| --- | --- | --- | --- | --- | --- | --- | --- | --- | --- | --- |
| Borho et al. (2020) | Y | Y | U | U | Y | Y | Y | Y | Y | *Include |
| Comtesse & Rosner (2019) | Y | Y | U | U | Y | N | Y | Na | Y | *Include |
| El Khoury (2018) | Y | Y | U | U | Y | Y | Y | Na | Y | *Include |
| Georgiadou et al. (2018) | Y | Y | U | U | Y | N | Y | Na | Y | *Include |
| Grochtdreis et al. (2020) | Y | Y | U | U | Y | N | Y | Na | Y | *Include |
| Haase et al. (2019) | Y | Y | U | U | Y | N | Y | Na | Y | *Include |
| Kaltenbach et al. (2018) | Y | Y | U | U | Y | N | Y | Y | Y | *Include |
| Löbel (2020) | Y | Y | U | U | Y | Y | Y | Na | Y | *Include |
| Nutsch & Bozorgmehr (2020) | Y | Y | U | U | Y | N | Y | Na | Y | *Include |
| von Haumeder et al. (2019) | Y | Y | U | U | Y | N | Y | Na | Y | *Include |
| Walther, Fuchs et al. (2020) | Y | Y | U | U | Y | Y | Y | Na | Y | *Include |
| Walther, Kröger et al. (2020) | Y | Y | U | U | Y | Y | Y | Na | Y | *Include |
| Winkler et al. (2019) | Y | Y | Y | U | Y | N | Y | Na | Y | *Include |

**Supplementary Table A3**. Validity of instruments used in the included studies.

| **First Author (year)** | **Measurement** | **DEP** | **GAD** | **PTSD** | **OTHERS** |
| --- | --- | --- | --- | --- | --- |
| Borho et al. (2020) | Self-report | PHQ-9  T1: α = 0.892  T2: α = 0.897 | GAD-7  T1: α = 0.907  T2: α = 0.918 | ETI  T1: α =0.961  T2: α =0.949 | Na |
| Comtesse & Rosner (2019) | Semi-structured clinical interview | PHQ-9  α = 0.83 | Na | PCL-5  α = 0.88 | TGI-SR: PGD, PCBD  Total PGD score: α = 0.92 |
| El Khoury (2018) | Self-report | MHI-18  α = U | Na | Na | MHI-18: Mental health  α = U |
| Georgiadou et al. (2018) | Self-report | PHQ-9  α = 0.70 | GAD-7  α = 0.92 | ETI  α = 0.95 | Na |
| Grochtdreis et al. (2020) | Computer-assisted face-to-face interview | Na | Na | Na | MCS: Mental health  α = U |
| Haase et al. (2019) | Self-report | Na | Na | Na | MIRIPS: Mental health  α = 0.926 |
| Kaltenbach et al. (2018) | Semi-structured clinical interview | PHQ-9  t0: α = 0.87  t6: α = 0.90  t12: α = 0.85 | Na | PSS-I  PCL-5:  t0: α = 0.89  t6: α = 0.95  t12: α = 0.90 | Na |
| Löbel (2020) | Computer-assisted face-to-face interview | Na | Na | Na | MCS: Mental health  α = U |
| Nutsch & Bozorgmehr (2020) | Computer-assisted face-to-face interview | PHQ-2  α = U | GAD-2  α = U | Na | Na |
| von Haumeder et al. (2019) | Self-report | Na | Na | PCL-5  α = 0.95 | Na |
| Walther, Fuchs et al. (2020) | Computer-assisted face-to-face interview | Na | Na | Na | PHQ-4: Psych. distress  α = 0.77 |
| Walther, Kröger et al. (2020) | Computer-assisted face-to-face interview | Na | Na | Na | RHS-13: Psych. distress  α = 0.91 |
| Winkler et al. (2019) | Self-report | HSCL-25  α = U | Na | PDS; HTQ  α = U | SOMA-Scale  α = U |

*Note:* *Na* = Not applicable, *U* = Unclear, *DEP* = Symptoms of depression, *GAD* = Symptoms of generalized anxiety disorder, *PCBD* = Symptoms of persistent complex bereavement disorder symptoms, *PDG* = Symptoms of prolonged grief disorder symptoms, *PTSD* = Symptoms of post-traumatic stress disorder, *T1* = Initial measurement, *T2* = Measurement after one and a half years, *t0* = Baseline measurement, *t6* = Measurement after six months, *t12* = Measurement after twelve months, *ETI* = Essen Trauma Inventory, *GAD-2* = Generalized Anxiety Disorder-2, *GAD-7* = Generalized Anxiety Disorder-7, *HTQ* = Harvard Trauma Questionnaire, *HSCL-25* = Hopkins-Symptom-Checklist 25, *MHI-18* = Mental Health Inventory, *MIRIPS* = Mutual Intercultural Relations in Plural Societies, *MCS* = Mental Health Component Summary Scale, *PCL-5* = Posttraumatic Stress Disorder Checklist-5, *PHQ-2* = Patient Health Questionnaire-2, *PHQ-4* = Patient Health Questionnaire for Depression and Anxiety, *PHQ-9* = Patient Health Questionnaire-9, *PDS* = Posttraumatic Diagnostic Scale, *PSS-I* = PTSD Symptom Scale - Interview Version, *RHS-13* = Refugee Health Screener-13, *SOMA-Scale* = SOMA-Scale of the Symtom-Checklist-90, *TGI-SR* = Traumatic Grief Inventory Self-Report Version
